# Based on the Beers Criteria 2019 Edition Over-the-Counter Drugs Risk Confirmation of Elderly Chinese

**DOI:** 10.1155/2021/5524551

**Published:** 2021-08-25

**Authors:** Yongyu Yang, Lu Zhang, Yamin Huang, Hangxing Huang, Shusen Sun, Jian Xiao

**Affiliations:** ^1^Department of Pharmacy, The Second People's Hospital of Beihai, Beihai, Guangxi, China 536000; ^2^Department of Pharmacy, Xiangya Hospital, Central South University, Changsha, Hunan, China 410008; ^3^National Medical Center for Geriatrics, Xiangya Hospital, Central South University, Laboratory for Rational and Safe Use of Elderly, Changsha, Hunan, China 410008; ^4^Department of Pharmacy Practice, College of Pharmacy and Health Sciences, Western New England University, Springfeld, MA 01119, USA; ^5^The Hunan Institute of Pharmacy Practice and Clinical Research, Changsha, Hunan 410008, China

## Abstract

**Objective:**

To explore OTC (over-the-counter drugs) in Chinese community pharmacies often causes ADE (adverse drug event) in elderly patients.

**Methods:**

Use the drugs in the Beers Criteria 2019 potentially inappropriate medication use (PIM) list as search terms. Search for drugs registered on the National Medical Products Administration of China website before December 2019 to determine the drugs containing PIM active ingredients and, then, search the Chinese OTC selection and conversion catalog database to determine it as OTC. Two databases are considered to be the same drug if they have the same drug composition.

**Results:**

The incidence of PIM in elderly patients in our community is relatively high, and the management of OTC may be related to risk factors. Statistics found that 71 OTC contained the Beers Criteria ingredients, including 65 chemicals and six Chinese patent medicines. Varieties of compound preparations accounted for 78.9% and cold medicines accounted for 47.9%.

**Conclusions:**

The high detection rate of the Beers Criteria in Chinese OTC suggests that medical practitioners in China, especially community pharmacists, should pay attention to the rational use of OTC in the elderly.

## 1. Introduction

Potentially inappropriate medication use (PIM) is considered a common cause of adverse health outcomes, and over-the-counter drugs (OTC) have contributed to the increased incidence of PIM [1]. A study of the use of OTC by the elderly in community pharmacies found that 95% of participants had at least one potential medication error, 80% of participants chose OTC, which may cause potential drug-drug interactions if taken with their current prescription drug treatment strategies [2]. American Geriatrics Society (AGS) Beers Criteria has now become an important evaluation standard for judging PIM in the elderly [3]. Hitherto, there is no study on the incidence of PIM in Chinese OTC based on the Beers Criteria, so the variety and quantity of the Beers Criteria active ingredients in Chinese OTC are unknown.

The city and hospital levels are independent risk factors that cause PIM in elderly patients in China [4]. The incidence of PIM was higher among elderly patients in rural outpatient clinics and retirement communities than in outpatient clinics in large cities. At the same time, a large number of studies have shown that the incidence of PIM in elderly hospitalized patients in China is 50%-70% [5–7]. A survey of outpatient prescriptions in 79 hospitals in China with 6 major cities based on the Beers Criteria 2012 stated that the incidence of PIM was 15.8%-23.0% [4]. According to the Beers Criteria 2019, a retrospective evaluation of 1,874 outpatients aged 65 years and older in first-tier cities in China, where the prevalence of PIM drugs was 35% [8], while the incidence of elderly people living in senior care communities was 39.0%-60.6% [9, 10]. 57% of elderly patients with chronic diseases taking prescription drugs and OTC were unsafe to take OTC [11]. The PIM and the adverse reaction (ADR) of elderly patients with chronic diseases in the Chinese community are extremely significantly correlated (*r* = 0.716, *P* < 0.01) [12]. Numerous relevant studies have shown that PIM drugs for elderly Chinese patients include proton pump inhibitors (PPIs) [10, 13–15], analgesic [9, 10, 12–14], and anticholinergic drugs [8, 10, 13, 14]. In the elderly patients with ADR caused by PIM, the PIM drugs include analgesic, anticholinergic drugs, PPIs, Chinese patent medicine, and compound preparations [12, 13].

OTC have the characteristics of safety in application, stable quality, accurate curative effect, convenient use, etc., and are generally targeted at mild diseases, low cost, convenient purchase, and extensive clinical use. Then, patients often buy OTC by themselves, thinking that OTC are safe and do not pay attention to potential drug risks, and it is more common for the elderly [16, 17]. Self-medication of elderly patients, including OTC, can easily lead to ADR [18, 19], PIM caused by the use of prescription drugs and OTC in elderly patients has attracted widespread attention [16, 17, 20]. A cross-sectional study conducted by the Health Counseling Center of a teaching hospital in Brazil, according to the Beers Criteria 2015, 80.5% of the elderly who self-medicate will purchase prescription and OTC without the supervision of professionals. 55.5% of people used the drugs on the PIM list recognized by the Beers Criteria, and OTC accounted for 52.6% of these drugs [18]. In the elderly at risk of major adverse drug event (ADE), more than 50% of the events involve OTC [2].

According to statistics, only 45% of residents in China have a certain awareness rate of OTC [21]. Xu et al. [22] analyzed 45 cases of ADR caused by OTC in the hospital, and OTC accounted for 12.6% of all ADRs, and people over 60 accounted for 24.4%. At present, PIM research in China is mostly limited to prescription drugs in hospitals or communities. It rarely involves self-medication of the elderly including OTC, especially some Chinese patent medicine and compound preparations [12, 23]. How to evaluate and prevent PIM in OTC for the elderly is a matter of concern.

Therefore, this study is aimed to use the Beers Criteria 2019 to count the varieties of PIM active ingredients in Chinese OTC and explore the risks of PIM in Chinese OTC according to the Beers Criteria 2019.

## 2. Methods

Use the drugs in the Beers Criteria 2019 PIM list as search terms. Search for drugs registered on the National Medical Products Administration of China website (https://www.nmpa.gov.cn/) before December 2019 to determine the drugs containing PIM active ingredients and, then, search the Chinese OTC selection and conversion catalog database to determine it as OTC. Two databases are considered to be the same drug if they have the same drug composition.

## 3. Results

A total of 71 kinds of OTC containing active ingredients of PIM drugs were screened, including 65 kinds of chemicals, 56 kinds of compound preparations, and 6 kinds of Chinese patent medicines. Chinese patent medicines are Chinese and Western medicine compound preparations containing chlorpheniramine, and phenobarbital and scopolamine tablets contain two kinds of PIM ingredients. The classification of drugs is based on China's “National Basic Medical Insurance, Work Injury Insurance and Maternity Insurance Drug Catalog” (2019 Edition) [24]; there are the most varieties of cold medicine and digestive system. There are 6 kinds of Chinese patent medicines, all of which are Chinese and western medicine compound preparations (See Table 1). In addition, cold medications accounted for 47.9%, of which PIM-containing active ingredients contained first-generation antihistamines, which accounted for 67.6% (48/71), and chlorpheniramine accounted for 39.4%, followed by digestive system drugs, antipyretic analgesics, and nonsteroidal anti-inflammatory drugs (NSAIDs) (See Table 2).

## 4. Discussion

Statistics show that there are 56 kinds of compound preparations and 34 kinds of cold medicines in OTC containing PIM active ingredients. Cold medicines of OTC are widely used in China. A survey by the China OTC Association shows that cold medicines have the highest proportion of self-medication among Chinese residents, accounting for 89% [21]. Among the 71 types of OTC we have counted, 47.9% are cold medicines, and six Chinese patent medicines are Chinese and Western compound preparations. Wang et al. [25] conducted a consolidated analysis of the relevant data in the “Annual Report on ADR Monitoring” issued by the National Medical Products Administration of China from 2009 to 2018, and the proportion of Chinese patent medicines was 10%-20%. In 2011, Wu et al. [26] conducted information data mining research on ADR of Chinese and Western compound preparations, collected and retrieved reports on ADR cases caused by Chinese and Western compound preparation published in China from 2007 to 2010, established a database, and used statistics Method analysis; the results show that cold medicines are the most reported ADR, with the elderly accounting for 29.51%. Cold medicines are mostly compound preparations and Chinese and Western compound preparations, which can easily cause ADR [22, 26] and severe ADR [19, 27]. Excessive medication has been the main cause of cold and Western compound preparations ADR or ADE [28]; the risk of ADR caused by Chinese and Western coordinated use or combination of drugs containing the same ingredients or with similar functions has attracted attention in China [29].

Statistics show that many OTCs contain ingredients with strong anticholinergic properties, including the first-generation antihistamines, chlorpheniramine, promethazine, diphenhydramine, and dimenhydrinate. In a small-sample statistic in China, PIM caused 15.5% of the first-generation antihistamines in ADR, and the proportion of compound preparations containing chlorpheniramine maleate was the highest [13]. The first-generation antihistamines are lipophilic drugs, which easily cross the blood-brain barrier and interact with many drugs. Due to anticholinergic side effects, the Beers Criteria included it on the PIM list, but still widely used in the elderly [30, 31]. The combination of prescription drugs and anticholinergic OTC in the elderly at the same time increases the burden of anticholinergic, leading to the risk of ADE and other negative consequences [15]. It is not recommended to use drugs with significant anticholinergic side effects [32].

The main category of ADR caused by OTC is NSAIDs [22], which are not only the most self-medication drugs for elderly patients [18, 19, 33, 34], but also the drugs most related to drug interactions [18]. NSAIDs may not only cause ADE due to drug interactions but also increase the risk of cardiovascular disease [35], causing dizziness, falls in the elderly, the occurrence or aggravation of cardiovascular disease [15], and increasing the risk of dementia and death in elderly patients [36]. In 2015, the Food and Drug Administration (FDA) issued a warning that NSAIDs increase the risk of heart disease and stroke [37]. On the other hand, NSAIDs can easily cause gastrointestinal system damage [19, 34], and the use of the elderly is more likely to cause serious gastrointestinal adverse reactions [38]. High-risk factors for PIM include age ≥75 years, oral corticosteroids, anticoagulants, or antiplatelet drugs [39, 40]. If there are no professionals to conduct medication evaluation, the use of NSAIDs by people with high-risk factors will increase the risk of ADE [18].

Elderly self-medication is universal [18, 41]. The important specific types of PIM for the elderly in prescription drugs and OTC are drug-drug and drug-disease interactions [40]. The causes of PIM in elderly patients in the Chinese community include not only multiple comorbidities and multiple medications but also many factors such as self-purchasing medications, long-term replacement medications, and repeated medications [42]. A variety of OTC contain PIM active ingredients. The use of OTC in elderly patients can easily lead to repeated medications and increase the risk of potential drug overdose and possible drug-drug interactions, leading to ADR [8, 16, 18]. The ADR of PIM is mostly a cumulative effect [18, 21], which can cause many adverse consequences in elderly patients. Compared with elderly patients without PIM, the risk of hospitalization in elderly patients with one type of PIM is 1.13 times, the risk of hospitalization with two types of PIM is 1.27 times, and the risk increases to 1.35 times with three types of PIM [43]. In addition, PIM in elderly patients can also lead to an increase in mortality. Compared with elderly patients without PIM, elderly patients with ≥1 PIM have an increased risk of death by 1.44 times [44].

Elderly chronic patients need more pharmacist intervention when using OTC [11]. As far as we know, Chinese elderly people lack reliable knowledge of multiple drugs [28, 32]. In China, a team of pharmacists and family doctors has little intervention in the rational use of OTC for elderly patients. How to ensure the safety of OTC for the elderly is a question worthy of further consideration for medical staff.

## 5. Conclusion

The high detection rate of the Beers Criteria in Chinese OTC suggests that medical practitioners in China, especially community pharmacists, should pay attention to the rational use of OTC in the elderly.

## Figures and Tables

**Table 1:** Varieties of OTC drugs containing PIM ingredients.

Indication PIM ingredient	Number of varieties (%)	Varieties
Cold medicines	34 (47.9)	
Chlorpheniramine	28 (39.4)	Paracetamol and Chlorphenamine maleate with three herb extracts capsules Paracetamol pseudoephedrine hydrochloride and chlorpheniramine maleate (capsules, chewable tablets, effervescent granules) Paracetamol, amantadine hydrochloride, Artificial cow-bezoar, and Chlorphenamine maleate granules Paracetamol, caffeine, Artificial cow-bezoar, and Chlorphenamine maleate (capsules, tablets) Paracetamol, caffeine, Guaifenesin, and Chlorphenamine maleate solution Paracetamol, caffeine, and Chlorphenamine tablets Paracetamol, caffeine, pseudoephedrine hydrochloride, and Chlorphenamine maleate capsules Composite Benorilate pseudoephedrine tablets Fēn kā má mǐn jiāonáng Paracetamol, pseudoephedrine hydrochloride, dextromethorphan hydrobromide, and Chlorphenamine maleate (capsules, tablets) Compound Paracetamol and dextromethorphan syrup Paracetamol, amantadine hydrochloride and Chlorphenamine maleate capsules Compound Paracetamol and Chlorphenamine maleate granules Compound Paracetamol and amantadine hydrochloride (capsules, granules, tablets) Compound Paracetamol and Sulfogaiaool Oral Solution Compound Asiatic Moonseed, Paracetamol, and Chlorphenamine maleate tablets Compound Paracetamol caffeine and pseudoephedrine hydrochloride capsules Granulae Zinci Gluconatis ET Ibuprofeni Compositae Compound Yinqiao and Paracetamol and Chlorphenamine maleate capsules Líng huáng ān kā mǐn piàn Psendoephedrine hydrochloride Chlorphenamine maleate and dextromethorphan Hydrobromide solution Paracetamol pseudoephedrine hydrochloride and Chlorpheniramine maleate granules Compound honeysuckle stem and aspirin tablets Diclofenac sodium enteric-coated tablets Guaifenesin, Pentoxyverine, citrate, and Chlorphenamine maleate tablets Guaifenesin, dextromethorphan hydrobromide, methylephedrine hydrochloride, and Chlorphenamine maleate syrup Compound Clorprenaline Hydrochloride tablets Compound Guaifenesine Pentoxyverine citrate and Chlorphenamine maleate syrup
Clemastine	1 (1.4)	Paracetamol Pseudophedrine sulfate and Clemastine Fumarate tablets
Ibuprofen	3 (4.2)	Ibuprofen and pseudoephedrine hydrochloride (tablets, dispersible tablets, granules) Granulae zinci cluconatis et ibuprofeni compositae Xīn bù piàn
Dimenhydrinate	2 (2.8)	Compound guaiacol potassium sulfonate oral solution Sulfogaiacol, Pentoxyverine, and promethazine granules
Antihistamine drug	7 (9.9)	
Chlorpheniramine	1 (1.4)	Chlorpheniramine maleate tablets
Dimenhydrinate	2 (2.8)	Diphenhydramine hydrochloride and menthol syrup Diphenhydramine hydrochloride tablets
Promethazine	1 (1.4)	Promethazine hydrochloride tablets
Cyproheptadine	1 (1.4)	Cyproheptadine Hydyochloride tablets
Triprolidine	1 (1.4)	Triprolidine hydrochloride capsules
Dimenhydrinate	1 (1.4)	Dimenhydrinate (tablets, Buccal tablets)
Digestive system drugs	15 (21.1)	
A. belladonna	12 (16.9)	Aluminum Magnesium and Belladonna tablets Compound Magnesium Trisilicate and sodium bicarbonate tablets Compound Belladonnate and Aluminium hydroxide (tablets, powder) Compound Glycyrrhiza, Bismuth, and Magnesium tablets Compound Aloe and vitamin U tablets Compound Aluminium hydroxide tablets Aluminium hydroxide gel Compound Corydalis tuber and Aluminium hydroxide tablets Compound Belladonnate and Aluminium hydroxide (tablets, powder) Medicated Leaven, Rhubarb, Sodium bicarbonate, and Aluminium hydroxide capsules Vitamin U, Belladonna, and Aluminium (capsules II, capsules, dispersible tablets) Vitamin U, Aluminium hydroxide, and Magnesium Trisilicate (capsules, tablets, tablets II)
Omeprazole	2 (2.8)	Omeprazole enteric-coated capsules Omeprazole magnesium enteric-coated tablets
Hyoscyamine	1 (1.4)	Raceanisodamine tablets
Antipyretic analgesics and NSAIDs	8 (11.3)	
Ibuprofen	2 (2.8)	Ibuprofen (tablets, dispersible tablets, capsules, syrup, Oral solution, Chewables tablets, Effervescent tablets) Ibuprofen arginine tablets
Diclofenac	1 (1.4)	Diclofenac potassium tablets
Ketoprofen	1 (1.4)	Ketoprofen (tablets, capsules)
Naproxen	1 (1.4)	Naproxen (tablets, capsules, sustained release capsules, sustained release tablets, enteric-coated microgranules capsules)
Diphenhydramine	1 (1.4)	Ān fēn lā míng piàn
Chlorzoxazone	1 (1.4)	Compound Chlorzoxazone tablets
Triprolidine	1 (1.4)	Paracetamol, Triprolidine hydrochloride, and pseudoephedrine hydrochloride tablets
Nervous system medicines	1 (1.4)	
Scopolamine Phenobarbital	1 (1.4)	Phenobarbital and scopolamine hydrobromide tablets
Chinese patent medicines	6 (8.5)	
Chlorpheniramine	6 (8.5)	Wéi C yín qiào kēlì, Wéi C yín qiào piàn Gǎnmào ān piàn Hāi kè píng jiāonáng Hāi tè líng jiāonáng, Hāi tè líng piàn, Hāi tè líng kēlì Bíyán kāng piàn Cāng é bíyán piàn

OTC: over-the-counter; PIM: potentially inappropriate medication; NSAIDs: nonsteroidal anti-inflammatory drugs. Some of the drugs were specific to China, and we translated the names of these drugs through Google translation.

**Table 2:** OTC pharmaceutical preparations and proportion.

Drugs	Preparation classes		Total	Proportion (%)
	Simple	Compound		
Anticholinergic drug			60	84.5
The first-generation antihistamines				
Chlorpheniramine	1	34		
Dimenhydrinate	1	2		
Diphenhydramine	2	1		
Promethazine	1			
Clemastine		1		
Cyproheptadine	1			
Triprolidine	1	1		
Antispasmodic				
A. belladonna		12		
Hyoscyamine	1	1		
Digestive system drugs			2	2.8
Omeprazole	2			
Analgesic			9	12.7
Oral nonselective COX inhibitors				
Ibuprofen	2	3		
Diclofenac	1			
Ketoprofen	1			
Naproxen	1			
Skeletal muscle relaxants				
Chlorzoxazone		1		
Total	15	56	71	100

OTC: over-the-counter; COX: cyclooxygenase.

## Data Availability

The data can be obtained from the corresponding author for appropriate reasons.
